# Social participation trajectories in late life and cognitive functioning – A sequence analysis based on Taiwan Longitudinal Study on aging

**DOI:** 10.1016/j.ssmph.2025.101821

**Published:** 2025-05-28

**Authors:** Yu-Tien Hsu, Hanno Hoven, Francine Grodstein, Tzu-Hung Liu, Chia-Rui Chang, Yu-Lin Hsieh, Jarvis T. Chen, Ichiro Kawachi

**Affiliations:** a Department of Social & Behavioral Sciences, Yale University School of Public Health, 60 College St, New Haven, CT, USA; b Institute for Occupational and Maritime Medicine (ZfAM), University Medical Centre Hamburg-Eppendorf (UKE), Seewartenstrasse 10, 22459, Hamburg, Germany; c Department of Sociology and Political Science, Centre for Global Health Inequalities Research (CHAIN), Norwegian University of Science and Technology (NTNU), Edvard Bulls veg 1, 7491, Trondheim, Norway; d Rush Alzheimer's Disease Center, Rush University Medical Center, 600 S. Paulina Street, Chicago, IL, USA; e Department of Family Medicine, Taipei Tzu Chi Hospital, Buddhist Tzu Chi Medical Foundation, No. 289, Jianguo Rd, Xindian District, New Taipei City, Taiwan; f School of Medicine, Tzu Chi University, No. 701, Sec. 3, Chung Yang Rd, Hualien City, Taiwan; g Department of Biostatistics, Harvard T.H. Chan School of Public Health, 677 Huntington Avenue, Boston, MA, USA; h Department of Medicine, Brigham and Women's Hospital, 75 Francis Street, Boston, MA, USA; i Harvard Medical School, 25 Shattuck St, Boston, MA, USA; j Department of Social & Behavioral Sciences, Harvard T.H. Chan School of Public Health, 677 Huntington Avenue, Boston, MA, USA; k Center for Biostatistics in AIDS Research, Harvard T.H. Chan School of Public Health, Boston, Massachusetts, USA

**Keywords:** Cognitive reserve, Social participation, Life course and developmental change, Life events and contexts, Sequence analysis

## Abstract

**Objective:**

Many studies have linked greater social participation to less cognitive decline with aging. Understanding of how social participation transitions can be associated with older adults’ cognitive function is crucial.

**Methods:**

We analyzed histories of social participation among middle-aged (50–64 years, n = 1900) and older (65+ years, n = 2500) participants in the Taiwan Longitudinal Study on Aging. We applied sequence analysis to define clustered social participation and work history in 1996, 1999, 2003, 2007, and 2011. We applied linear regression models for the associations between cluster membership and cognitive function in 2015, measured with the Short Portable Mental Status Questionnaire (SPMSQ). We used multinomial regression analysis to explore the factors related to belonging to a cluster.

**Results:**

In middle-aged adults, participation in multiple social activities was associated with higher cognition scores. Working and multiple activity participation among older adults were associated with higher scores. More active patterns of social participation were found for men, at younger ages, and among non-urban residents.

**Discussion:**

Our study findings support the value of social engagement and work involvement for healthy cognitive aging. Additionally, we identified subgroups that were more likely to be socially engaged.

## Highlights

• Sequence analysis reveals distinct social participation patterns linked to cognitive function.
• Multiple social activities and continued work predict better cognitive outcomes in later life.
• Gender gaps exist in work and social participation among older Taiwanese adults.

## 1 Introduction

Social participation is a construct that captures an individual's involvement in activities that provide interactions with others (Levasseur, Généreux, et al., 2015). It encompasses interpersonal interactions such as face-to-face time with neighbors or friends (Zettel-Watson & Britton, 2008) and engagement in formal organizations such as political parties or religious groups (Aroogh & Shahboulaghi, 2020). Social participation is shaped by individual factors such as gender (Avlund et al., 2004; Kishimoto et al., 2013). For example, studies found that older women may prefer to join groups offering volunteering opportunities (Gilmour, 2012), while men tended to participate in exercise-based groups (Kanamori et al., 2015). Social participation is also influenced by physical and social infrastructure, e.g., urban design, access to transportation, and facilities or amenities for socializing. Therefore, people may face different sets of facilitators or barriers to social participation.

Social participation is related to cognitive aging through several plausible pathways. First, social participation engages people in cognitively stimulating and mental activities that may help maintain brain function (Lafortune & Brayne, 2017). Second, social participation can expand or maintain an individual's social network, engaging elderly people in meaningful social roles and preventing stress-related neurological damage (Kremen et al., 2012). Third, engaging in social activities could simultaneously increase older adults' physical activity, which is associated with greater maintenance of brain structure (Sexton et al., 2016) and reduced risk of vascular risk factors for cognitive decline (Berlin & Colditz, 1990).

There are gaps remaining in the literature. For example, most definitions of social participation do not consider the level of involvement or interaction with others when participating in social activities or the goals of the activities. Using a framework such as Levasseur's social participation model (Levasseur et al., 2010) could help researchers conceptualize and select essential activities related to social participation. Moreover, many existing studies fail to consider the complete history of social participation throughout the aging process. As individuals may modify their activities over time, these changes could have varying effects on cognitive health maintenance. In addition, the literature remains limited in non-Western societal contexts (Evans et al., 2019); for example, education and gender disparities in social participation have rarely been studied in Asia.

Taiwan is a rapidly aging society, with projections indicating it will become a "super-aged society" in 2025 when more than 20 % of its population will be 65 or older (Everington; National Development Council). This demographic transition is occurring at one of the fastest rates in the world, driven by consistently low fertility rates and increasing life expectancy (Hsueh). In 2024, 19.2 % of Taiwan's population was already aged 65 and older, with forecasts suggesting this share could reach 38.4 % by 2050 (United Nations et al., 2020). Therefore, promoting social participation may represent an important strategy for healthy aging in Taiwanese society. Cultural values in Taiwan are rooted in Confucianism, emphasizing maintaining strong family ties, social connections, and respect for elders. Nevertheless, older women in Taiwan often have weak labor force participation (Yu), low education levels (The Ministry of Health and Welfare), and other barriers to social engagement. In the current study, we sought to (1) define clusters of social participation and work status with similar patterns in middle-aged and older Taiwanese adults, (2) examine the associations between each cluster pattern of social participation over time with cognitive function in later life, and (3) explore sociodemographic factors related to membership in clusters.

## 2 Methods

### 2.1 Participants

The data for this study were drawn from the Taiwan Longitudinal Study in Aging (TLSA), a nationally representative, population-based cohort study initiated by the Health Promotion Administration, Ministry of Health and Welfare in Taiwan in 1989. This longitudinal study employs a multi-stage stratified systematic sampling approach to ensure demographic representativeness across Taiwan's diverse regions. Response rates were close to 100 percent at multiple subsequent waves (Martin et al., 2011). Our analysis utilized survey data spanning from 1996 to 2015, as cognitive function assessment was incorporated beginning with the 1996 wave. Follow-up surveys were administered in 1999, 2003, 2007, 2011, and 2015, with detailed participation rates documented in Supplementary Table 1. The original study sample was 6473. For our analysis, we excluded participants whose baseline (1996) cognitive scores fell at or below the 30th percentile (n = 2071) to focus on those without significant cognitive impairment at baseline. We further excluded 4 participants with data inconsistencies in cognitive function scores or a number of grandchildren. The final analytic sample comprised 4400 participants who were followed for up to 19 years, providing a robust longitudinal dataset for examining trajectories of social participation and cognitive function. All data used in this study were de-identified and obtained through the Ministry of Health and Welfare, Taiwan, following their established protocols for protecting participant privacy. The details of the cohort and study design of TLSA are described on the TLSA website and in other sources (Liang et al., 2010; Taiwan Longitudinal Study on Aging).

### 2.2 Measures

#### 2.2.1 Independent variable: social participation and work histories clusters

The independent variable for the primary analysis was the categorical variable of an individual's membership in distinct clusters of social participation and work histories created through sequence analysis and optimal matching techniques. Individuals' social participation and working status were assessed in every survey wave, resulting in a history of social participation and work involvement from 1996 to 2011 (a total of five waves) that covered 15 years of participants' social lives. The details of the categorization of social activities and the formation of social participation and working sequence for the participants were summarized in Appendix A.

**Fig. 1:**
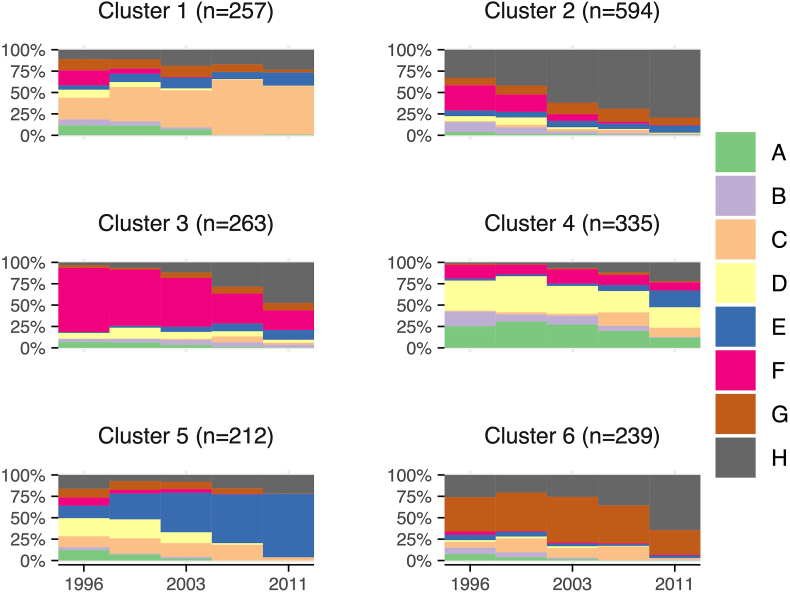
Clusters of social participation histories, middle-aged subgroup (age 50–64 in 1996). (N = 1900)∗ ∗Note: Social participation state A: helping, working, and socializing; State B: helping and working; State C: helping and socializing; State D: working and socializing; State E: socializing only; State F: working only; State G: helping only; State H: did not involve in any of the social participation types.

**Fig. 2:**
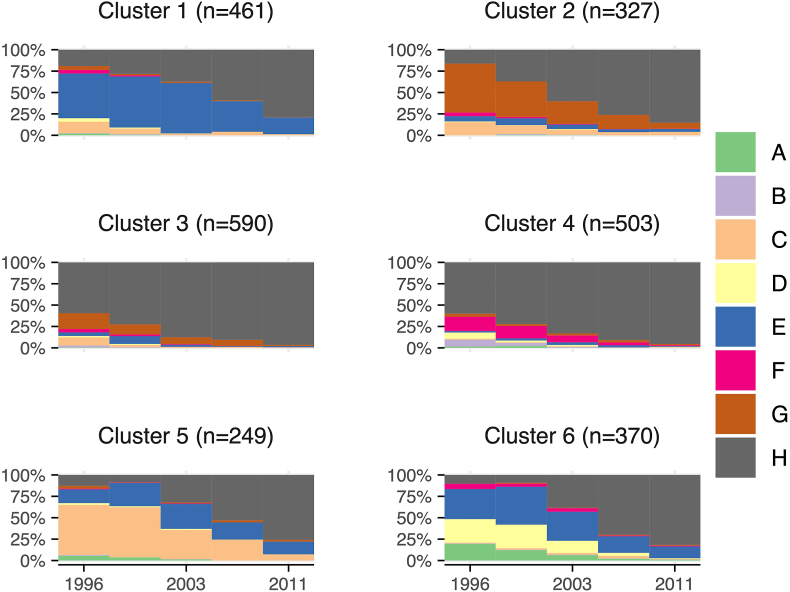
Clusters of social participation histories, older subgroup (age 65+ in 1996). (N = 2500) ^†^ **^†^**Note: Social participation state A: helping, working, and socializing; State B: helping and working; State C: helping and socializing; State D: working and socializing; State E: socializing only; State F: working only; State G: helping only; State H: did not involve in any of the social participation types.

We identified six clusters in both subgroups and chose the clusters with the lowest social participation pattern in each subgroup as the reference group (Cluster 2 in the middle-aged subgroup and Cluster 3 in the older subgroup). Cluster compositions are illustrated in Fig. 1, Fig. 2, and descriptions for each cluster are presented in Table 2, Table 3. In the middle-aged subgroup, Cluster 1 (socializing & helping) represents participants who transitioned to socializing and helping activities over time. Cluster 2 (low social participation) represents participants who started in the working dominant state but transitioned to a low social participation state. Cluster 3 (working) predominantly comprised participants who were working. Cluster 4 (active in multiple states) captured participants who were consistently involved in multiple types of social participation throughout the study period. Cluster 5 (socializing) represents participants who transitioned to the “socializing only” state over time. Cluster 6 (helping) represents participants who were stably involved in “helping only” activities over time.

**Table 1:** Sociodemographic characteristics of the study participants (N = 4400).

Sociodemographic Characteristics	Total	Older Subgroup (Age 65+ in 1996)	Middle-aged subgroup (Age 50–64 in 1996)
**No. Participants**	4400	2500 (100 %)	1900 (100 %)
**Age**			
50–54 years	646 (14.7 %)	–	646 (34.0 %)
55–59 years	701 (15.9 %)	–	701 (36.9 %)
60–64 years	553 (12.6 %)	–	553 (29.1 %)
65–69 years	861 (19.6 %)	861 (34.4 %)	–
70–74 years	656 (14.9 %)	656 (26.2 %)	–
75–79 years	713 (16.2 %)	713 (28.5 %)	–
80 years or above	270 (6.1 %)	270 (10.9 %)	–
**Education level**			
No education	1294 (29.4 %)	855 (34.2 %)	439 (23.1 %)
Elementary school	2047 (46.5 %)	1084 (43.4 %)	961 (50.6 %)
Middle/High school	789 (17.9 %)	410 (16.4 %)	379 (19.9 %)
College or above	272 (6.2 %)	151 (6.0 %)	121 (6.4 %)
**Ethnicity**			
Han	2918 (66.3 %)	1466 (58.6 %)	1452 (76.4 %)
Mainlander	717 (16.3 %)	393 (15.7 %)	324 (17.1 %)
Hakka	705 (16.0 %)	606 (24.2 %)	99 (5.2 %)
Others	51 (1.2 %)	27 (1.1 %)	24 (1.3 %)
Missing	9 (0.2 %)	8 (0.3 %)	1 (0.1 %)
**Marital Status**			
Married/Partnered	2582 (58.7 %)	1199 (48.0 %)	1383 (72.8 %)
Widowed/Divorced	1818 (41.3 %)	1301 (52.0 %)	517 (27.2 %)
**Urbanicity**			
Stable urban region	1372 (31.2 %)	791 (31.6 %)	581 (30.6 %)
Township	376 (8.5 %)	245 (9.8 %)	131 (6.9 %)
Rural region	940 (18.9 %)	529 (21.2 %)	302 (15.9 %)
Move to township or rural regions	658 (15.0 %)	332 (13.3 %)	326 (17.2 %)
Move to township or urban region	1124 (25.5 %)	581 (23.2 %)	543 (28.6 %)
Missing	39 (0.9 %)	22 (1.0 %)	17 (0.9 %)
**Medical Condition**			
Hypertension	2427 (55.2 %)	1418 (56.7 %)	1009 (53.1 %)
Diabetes	1119 (25.4 %)	609 (24.4 %)	510 (26.8 %)
Cancer	335 (7.6 %)	181 (7.4 %)	154 (8.1 %)
Baseline mean CESD Score (SD)	7.1 (4.4)	7.4 (4.6)	6.7 (4.1)

**Table 2:** Distribution, description, and dominant states of social participation and working clusters, middle-aged subgroup (age 50–64 in 1996). (N = 1900).

Cluster	Label	Number of participants (%)	Description	Dominant States
**1**	Socializing and helping	257 (13.5 %)	Increased socializing and helping states.	Socializing only; Helping and socializing.
**2**	Low social participation	594 (31.3 %)	Transitioning from working to low social participation state.	Low social participation; Helping only.
**3**	Working	263 (13.8 %)	Working state dominant, transitioning into low social participation, helping, or socializing over time.	Working only.
**4**	Active in multiple states	335 (17.6 %)	Stable socializing, working, and helping states over time.	Helping, socializing, and working; Working and socializing
**5**	Socializing	212 (11.2 %)	Transitioning into socializing state.	Socializing only.
**6**	Helping	239 (12.6 %)	Stable helping state over time.	Helping only.
**Total**	1900 (100 %)

**Table 3:** Distribution, description, and dominant states of social participation and working clusters, older subgroup (age 65+ in 1996). (N = 2500).

Cluster	Label	Number of participants (%)	Description	Dominant States
1	Socializing	461 (18.4 %)	Socializing only state dominant, decreasing over time.	Socializing only; low social participation.
2	Helping	327 (13.1 %)	Helping only state dominant over time.	Helping only.
3	Low social participation	590 (23.6 %)	Increased low social participation state; decreased helping only state.	Low social participation.
4	Working & low social participation	503 (20.1 %)	Low social participation state and working only state dominant.	Low social participation; working only.
5	Helping & socializing	249 (10.0 %)	Helping and socializing state and socializing only state dominant, decreasing over time.	Helping and socializing; socializing only.
6	Active in multiple states	370 (14.8 %)	Socializing only state dominant, stable over time; working and socializing state and helping, working, and socializing state dominant, decreasing over time.	Socializing only; working and socializing state; helping, working, and socializing state
Total		2500 (100 %)		

We identified six clusters for the older subgroup. Cluster 1 (socializing) represents participants who did socializing activities but transitioned to low social participation over time. Cluster 2 (helping) featured a group of respondents who were stably involved in helping activities over time. Cluster 3 (low social participation) captured participants who were inactive in any social participation. Cluster 4 (working & low social participation) represents a group working only or in a low social participation state. Cluster 5 (helping & socializing) represents people who transitioned to low social participation over time but were stably engaged in helping. Cluster 6 (active in multiple states) represents active participants in various social participation or working states.

#### 2.2.2 Dependent variable: cognitive function score

Our dependent variable was the cognitive score in 2015, measured by the 10-item Short Portable Mental Status Questionnaire (SPMSQ) score (Pfeiffer et al., 1984). The possible score ranges from 0 to 10. Studies based on the older Taiwanese population reported strong internal consistency reliability (Lee et al., 2022). The Cronbach's alpha of the SPMSQ in this sample is 0.91.

#### 2.2.3 Covariates

We controlled for medical history (cancer, diabetes, and stroke), depressive symptoms measured by the Center for Epidemiological Studies Depression (CESD) scale (0–30 points), sociodemographic variables including age (categorized as 50–59, 60–69, 70–79, and 80 years or above), sex (male vs. female), educational attainment (no formal education, elementary school graduate, middle or high school graduate, college graduate or above), urbanicity of residence (urban region, township, rural region, moving to a more urban region, or move to a more rural region), health behaviors (smoking, drinking, betel nut use, regular exercise), and family conditions such as marital status (married/partnered vs. widowed, divorced or separate) and number of grandchildren.

#### 2.2.4 Statistical models

There are several steps in our analytical process. First, we applied sequence analysis (Abbott, 1995; Aisenbrey & Fasang, 2010) and optimal matching techniques (Halpin, 2012; Studer & Ritschard, 2016) to identify and group people with similar patterns of social participation histories. Second, we performed multinomial logistic regression to identify characteristics associated with cluster memberships. Lastly, we ran linear regression models with inverse probability of selection weights to examine the association between social participation clusters and cognitive function in 2015. We stratified our analysis into two sub-groups using the age cutoff of 65 (middle-aged and older subgroups) since it is the legal retirement age for pension eligibility in Taiwan during the data collection period. The rationale for doing the subgroup analysis is that age is a crucial factor affecting people's social participation and employment status.

### 2.3 Sequence analysis & optimal matching

We selected sequence analysis with optimal matching as the analytical approach, as it captures the complex patterns of social participation and work experiences over time. This method's key advantage lies in its ability to account for the ordering, transitions, and combinations of different types of social engagement throughout participants' life trajectories (Abbott & Tsay, 2000; Halpin & Cban, 1998). Unlike cross-sectional approaches that provide only snapshots of participation or aggregated measures that lose temporal information, sequence analysis preserves the dynamic nature of social and occupational patterns that may critically influence cognitive outcomes.

We linked each participant's social participation and working status across five survey waves to form a sequence. We then applied the optimal matching algorithm to group participants with similar patterns to compare each participant's social participation and working trajectory with all other trajectories by using the *WeightedCluster* package (Studer, 2013) in R. The optimal matching technique calculates the "distance" between different state sequences by determining the minimum number of operations (substitutions, insertions, or deletions) needed to transform one sequence into another, then groups similar sequences together using hierarchical clustering methods (Abbott & Tsay, 2000). This methodological distinction is essential because while states provide a cross-sectional snapshot of participation patterns, clusters reveal the longitudinal trajectories of how these patterns evolve over time. Sequence analysis and optimal matching techniques have been widely applied to study lifecourse transitions, such as employment (Hoven et al., 2018) and family formation (Wahrendorf et al., 2018). The novelty of our approach is in extending these methods to examine the social participation histories of older individuals. We quantitatively and qualitatively assessed the identified clusters by examining whether they have sensible interpretations (see Supplementary Tables 2 and 3 for the details).

### 2.4 Predictors for cluster membership

After obtaining the clustering results, we conducted multinomial logistic regression to assess whether age, education, marital status, urbanicity of residence, gender, diagnosis of diabetes or cancer, depression score, smoking, alcohol drinking, betel nut use, and exercise predict cluster membership.

### 2.5 Association between cluster membership and cognitive functioning in 2015

We examined the association between cluster membership (using the low social participation cluster as the reference group) and cognitive functioning in 2015 through linear regression while controlling for baseline covariates, including marital status, educational attainment, urbanicity of residence, medical diagnosis (cancer, hypertension, diabetes), CESD score, health behaviors (smoking, drinking, betel nut use, regular exercise), and number of grandchildren. We applied inverse probability of censoring weights (IPCW) in both regression models to address concerns about differential loss to follow-up (see Supplement Table 3). The details of the weighting method are presented in the Appendices. We conducted subgroup analyses by age, sex, and urbanicity to test whether the association between social participation pattern and cognitive function differed by socioeconomic factors.

### 2.6 Missingness in social participation and working states

The loss-to-follow-up and missingness of baseline sociodemographic variables, social participation, and working data in each wave were summarized in Supplementary Tables 4 and 5 We used participants’ previous non-missing social participation and working states, education attainment, age, gender, and urbanicity of residence to impute the missingness of social participation and working states in the following waves by the random forest imputation method. The multiple imputations were conducted by the “mice” package in R (Van Buuren & Groothuis-Oudshoorn, 2011). We generated 40 imputed datasets and performed the analyses described above; then, we combined the results across the imputations. All the analyses were conducted in R (version 4.3.1; R Development Core Team).

## 3 Results

### 3.1 Participants characteristics

Table 1 summarizes the sociodemographic characteristics of the study participants by subgroup. There were 2500 and 1400 participants in the older and middle-aged subgroups, respectively. Notably, nearly one-third of the participants did not receive any formal education (34.2 % for the older and 23.1 % for the middle-aged subgroup), and almost half of the participants had a level of educational attainment at the elementary school level (43.4 % for the older and 50.6 % for the middle-aged subgroup). Around half of the participants in the older subgroup experienced widowhood or divorce during the study period, while less than a third of participants in the middle-aged subgroup experienced widowhood or divorce during the same period.

### 3.2 Sociodemographic predictors of cluster membership

The multinomial logistic regression model (Supplementary Table 6) showed that in the middle-aged subgroup, women were less likely to belong to cluster 3 (Working) (Relative Risk Ratio (RRR) = 0.29, 95 % C.I. = 0.16–0.54, p < 0.001) or cluster 4 (Active in multiple states) (RRR = 0.11, 95 % C.I. = 0.06–0.20, p < 0.001) than cluster 2 (Low social participation) compared to men. Interestingly, there were striking urban-rural differences in social participation patterns among the middle-aged subgroup. Residents of townships and rural regions demonstrated significantly higher engagement in active social participation compared to their urban counterparts. Specifically, township residents were 2.5 times more likely (RRR = 2.50, 95 % C.I. = 1.24–5.07, p = 0.011) and rural residents were 4 times more likely (RRR = 4.06, 95 % C.I. = 2.33–7.09, p < 0.001) to belong to Cluster 4 (Active in multiple states) than Cluster 2 (Low social participation). This urban-rural gradient was also evident in work-focused cluster membership, with rural residents being nearly 4 times more likely to engage in work and socializing patterns.

Similarly, women in the older subgroup were less likely to belong to the cluster with working as the dominant state (Cluster 4, working & low social participation) (RRR = 0.39, 95 % C.I. = 0.24–0.64, p < 0.001) and Cluster 6 (active in multiple states) (RRR = 0.19, 95 % C.I. = 0.11–0.32, p < 0.001) than Cluster 2 (low social participation) compared to men. Furthermore, urban-rural differences became even more pronounced in the older subgroup. Both township (RRR = 1.84, 95 % C.I. = 1.08–3.11, p = 0.023) and rural residents (RRR = 2.04, 95 % C.I. = 1.27–3.28, p = 0.003) were approximately twice as likely to maintain working patterns (Cluster 4) compared to urban residents. The rural advantage extended to pro-social activities, with township residents 2.6 times more likely (RRR = 2.61, 95 % C.I. = 1.35–5.05, p = 0.004) and rural residents 4.3 times more likely (RRR = 4.30, 95 % C.I. = 2.45–7.57, p < 0.001) to engage in helping and socializing activities (Cluster 5) (see 78).

### 3.3 Associations between social participation cluster membership and late-life cognitive function

Table 4 summarizes the mean (SD) SPMSQ score at the baseline across clusters. In the middle-aged subgroup, mean SPMSQ scores range from 5.0 in Cluster 2 (Low social participation) and Cluster 5 (Socializing) to 5.6 in Cluster 4 (Active in multiple states). Quantile regression showed no statistically significant differences in median score across subgroups (all p-values = 1.000). In the older subgroup, mean scores range from 7.3 in Cluster 4 (Working & low social participation) to 7.7 in Cluster 1 (Socializing), Cluster 5 (Helping & Socializing), and Cluster 6 (Active in multiple states). Quantile regression did not find statistically significant differences in median score across clusters (all p-values = 1.000). This suggested a relatively homogeneous distribution of cognitive function at the median level across different social participation patterns.

**Table 4:** Baseline cognitive function across clusters.

**Middle-Aged Subgroup**			
**Cluster**	**Number of participants (%)**	**Cluster Label**	**Baseline Mean SPMSQ Score (SD)**

**1**	257 (13.5 %)	Socializing and helping	5.3 (1.9)
**2**	594 (31.3 %)	Low social participation	5.0 (1.8)
**3**	263 (13.8 %)	Working	5.4 (1.9)
**4**	335 (17.6 %)	Active in multiple states	5.7 (2.1)
**5**	212 (11.2 %)	Socializing	5.0 (2.0)
**6**	239 (12.6 %)	Helping	5.2 (1.9)
**Total**	1900 (100 %)		5.2 (1.9)

**Older Subgroup**			
**Cluster**	**Number of participants (%)**	**Cluster Label**	**Baseline Mean SPMSQ Score (SD)**

1	461 (18.4 %)	Socializing	7.7 (1.1)
2	327 (13.1 %)	Helping	7.6 (1.4)
3	590 (23.6 %)	Low social participation	7.5 (1.4)
4	503 (20.1 %)	Working & low social participation	7.4 (1.3)
5	249 (10.0 %)	Helping & socializing	7.7 (1.4)
6	370 (14.8 %)	Active in multiple states	7.6 (1.2)
Total	2500 (100 %)		7.5 (1.3)

Note: Cluster numbers and characteristics vary between age groups as they represent distinct empirically derived patterns from our sequence analysis. Reference categories are "Low social participation" in both subgroups (Cluster 2 for middle-aged, Cluster 3 for older adults).

In the middle-aged subgroup (aged 50–64 at baseline), using Cluster 2 (Low social participation) as the reference category, participants in Cluster 3 (Working) demonstrated significantly higher cognitive scores (mean difference = 0.52, 95 % C.I. = 0.03–1.01, p = 0.036), as did those in Cluster 4 (Active in multiple states) (mean difference = 0.58, 95 % C.I. = 0.09–1.06, p = 0.020). Notably, clusters with a predominant single form of social participation – Cluster 1 (Socializing & helping) (p = 0.051), Cluster 5 (Socializing) (p = 0.505), and Cluster 6 (Helping) (p = 0.809) did not show statistically significant associations with cognitive function (Table 5).

**Table 5:** Association between social participation clustering and score on the Short Portable Mental Status Questionnaire using OLS regression and censoring weights^a^.

**Cognitive Score (in 2015)**			
**Middle-aged subgroup (age 50–64 in 1996). (N** = **1900)**			
**Cluster**	**Mean Difference**	**95 % C.I.**	**p-value**

Cluster 2: Low social participation (Ref)	–	–	–
Cluster 1: Socializing & helping	0.49	(-0.01 – 0.98)	0.051
Cluster 3: Working	**0.52**	**(0.03**–**1.01)**	**0.036**
Cluster 4: Active in multiple states	**0.58**	**(0.09**–**1.06)**	**0.020**
Cluster 5: Socializing	0.17	(-0.33 – 0.67)	0.505
Cluster 6: Helping	0.06	(-0.46 – 0.59)	0.809

**Older subgroup (age 65** + **in 1996). (N** = **2500)**			
**Cluster**	**Mean difference**	**95 % C.I.**	**p-value**

Cluster 3: Low social participation (Ref)	–	–	–
Cluster 1: Socializing	0.32	(-0.48 – 1.12)	0.430
Cluster 2: Helping	0.39	(-0.78 – 1.55)	0.517
Cluster 4: Working & low social participation	**2.33**	**(1.31**–**3.36)**	< **0.001**
Cluster 5: Helping & socializing	**1.15**	**(0.05**–**2.26)**	**0.042**
Cluster 6: Active in multiple states	−0.32	(-1.24 – 0.60)	0.496

a Adjusted for marital status, educational attainment, urbanicity of residence, medical diagnosis (cancer, hypertension, diabetes), baseline CESD score, health behaviors (smoking, drinking, betel nut use, regular exercise), and number of grandchildren. Statistically significant estimates in bold.

Among the older subgroup (aged 65+ at baseline), with Cluster 3 (Low social participation) as a reference, the most substantial cognitive advantage was observed in Cluster 4 (Working & low social participation), revealing a significant mean difference of 2.33 points (95 % C.I. = 1.31–3.36, p < 0.001). Cluster 5 (Helping & socializing) also demonstrated significantly higher cognitive function (mean difference = 1.15, 95 % C.I. = 0.05–2.26, p = 0.042), while clusters with singular participation – Cluster 1 (Socializing) (p = 0.430) and Cluster 2 (Helping) (p = 0.517) showed no significant associations (Table 5).

Subgroup analyses revealed substantial heterogeneity in the association between social participation clusters and cognitive function. Among middle-aged adults, the cognitive benefits of working (Cluster 3) and active participation in multiple states (Cluster 4) were primarily driven by the youngest participants (aged 50–54) (Supplementary Table 8) and by women, with no significant associations observed among men (Supplementary Table 9) or any urbanicity subgroups (Supplementary Table 10). In the older subgroup, the strong protective association of working (Cluster 4, mean difference = 2.33 in main analysis) was most pronounced among those aged 70–74 (mean difference = 2.75) and 75+ (mean difference = 3.96) (Supplementary Table 11). Gender differences were evident, with older women experiencing cognitive benefits across multiple participation patterns (socializing, helping, and working), while older men benefited primarily from working patterns only (Supplementary Table 12). Urbanicity analyses revealed that urban older adults showed benefits across nearly all participation patterns compared to low participation, while rural older adults demonstrated significant cognitive benefits, specifically from socializing and working patterns. In contrast, township residents showed negative associations between socializing and cognitive function (Supplementary Table 13).

## 4 Discussion

Our study found that clusters characterized by consistent participation in multiple social activities and extended work engagement were associated with higher cognitive function in late life. These findings align with the cognitive enrichment theory, which posits that social engagement and continued work stimulate cognitive capacity in older adults, potentially protecting against cognitive decline (Milgram et al., 2006). Moreover, our findings strongly support the Cognitive Reserve Theory, showing that lifetime experiences, including social engagement and continued work, build resilience against age-related cognitive decline by enhancing neural networks and cognitive processing capacity (M Tucker & Stern, 2011). Specifically, our sequence analysis revealed that individuals with sustained patterns of diverse social participation—including socializing, helping activities, and extended work engagement—demonstrated significantly better cognitive outcomes than those with limited or inconsistent participation patterns. Our approach to examining social participation trajectories over time provides empirical evidence that multiple dimensions of social participation, its dynamic nature, diversity of activities, and duration of engagement across the life course, are linked to cognitive function.

Notably, not every cluster with active social participation or working histories was associated with a higher level of cognitive function in our analysis. Clusters dominated by solely helping or socializing activities were not associated with better cognitive function. This finding aligns with some studies that found not all types of social activities confer equal benefits. For instance, Hsu (2007) demonstrated that simple participation in routine social gatherings showed no significant association with cognitive function, while more comprehensive forms of social engagement encompassing recreational and cognitively challenging activities were associated with slower cognitive decline (Hsu, 2007). This selective benefit pattern may be explained by differences in cognitive demand and social network diversity across activity types. Previous research suggests that the cognitive benefits of social activities depend on their complexity and the level of mental stimulation they provide (Kelly et al., 2017). Work engagement, for instance, typically requires problem-solving, decision-making, and adaptation to changing circumstances, offering more intensive cognitive stimulation than routine social interactions or helping activities. Furthermore, work environments often expose individuals to diverse social networks and novel challenges, providing greater cognitive enrichment than activities confined to familiar social circles (Vance et al., 2016). Social network characteristics, including size and diversity, also influence cognitive outcomes. Research indicates that while broader and more varied social networks correlate with enhanced cognitive function, the magnitude of these benefits depends on both the nature and quality of social connections (Kim et al., 2022). Specifically, regular and meaningful interactions demonstrate stronger associations with cognitive health compared to superficial or routine social contacts. The finding that involvement solely in socializing or helping activities may not provide cognitive protection as robust as that of continued work suggests that the quality and diversity of cognitive stimulation matter more than mere social contact.

Our findings from Taiwan provide insights into how social participation patterns influence cognitive function in a society where social relationships are deeply embedded in cultural values emphasizing family connections, filial piety, and community harmony. This aligns with research among Chinese populations showing that tight-knit family relationships and intergenerational support, upward (from children to parents) and downward (grandparental caregiving), significantly benefit cognitive health, highlighting the central role of family-based social engagement (Li et al., 2021). Cross-cultural research indicates that perceptions of aging and the nature of social participation could differ between Eastern and Western societies (Löckenhoff et al., 2009), potentially influencing how different participation patterns contribute to cognitive reserve. For instance, the protective effect of engaging in multiple types of social participation, including helping, socializing, and working, observed in our study, may reflect the cultural emphasis on productive contribution to family welfare in Taiwanese society (Wang et al., 2019). In comparison, studies from Western countries have sometimes found stronger associations between formal organizational participation (like volunteering) and cognitive benefits (Ailshire & Carr, 2021). Despite these cultural differences in participation patterns, our findings align with studies that found protective relationships between social participation and cognitive health, showing the universal effect of social engagement across societies/cultures (Bourassa et al., 2017).

Our analyses yielded several new insights on older adults’ social participation behavior, especially for the Taiwanese and East Asian societies. We found substantial proportions of participants in both younger and older subgroups in the low social participation state (state H), meaning they were not involved in working, socializing, or helping others. For the younger subgroup, state H participants increased from 20 % to 35 % between baseline and final wave (Supplementary Table 14); for the older subgroup, they rose from 35 % to 70 % (Supplementary Table 15). Part of the decreasing social participation could be explained by aging. Previous studies reported that older age is a key factor in decreasing social participation (Bowling & Stafford, 2007; Huxhold et al., 2014; Naud et al., 2019). The younger subgroup participants were still working or had just reached the legal retirement age (i.e., 65 years old) in the earlier waves of the study period. However, as the participants aged, their social participation declined. The pattern was opposite compared to previous findings from the European countries and the United States (Cornwell et al., 2008; Kalbarczyk & Łopaciuk-Gonczaryk, 2022), especially among women (Kalbarczyk & Łopaciuk-Gonczaryk, 2022) and more educated people (Agahi & Parker, 2005; Butrica et al., 2009; Hank & Stuck, 2008; Kalbarczyk & Łopaciuk-Gonczaryk, 2022). However, other studies conducted in Asian societies reported a similar pattern to that we found (Ang, 2018; Gao et al., 2018). A study in Japan found that 20–50 % of older adults did not engage in any social activities (Ejiri et al., 2019). Another study conducted in South Korea reported that only 3.9 % of the older adults participated in volunteer work, and 4.5 % of them went to social clubs (Lee & Choi, 2020). An explanation for this limited participation phenomenon could be attributed to the centrality of family in Asian cultures, where social relationships are hierarchically organized with family obligations taking precedence over external social activities (Cheung & Kwan, 2000; Kikuzawa, 2006; Li et al., 2006). In Confucian-influenced societies like Taiwan, older adults often derive their primary source of social support and daily interaction from family members (Chen et al., 2015; The 2018 Aging Readiness, 2019), which may reduce their perceived need for external social engagement. This cultural pattern contrasts with Western societies, where peer relationships and community involvement often remain essential sources of social connection throughout the life course (Cornwell et al., 2008). These cultural factors may contribute to the observed patterns of low social participation among older adults in Asian societies, highlighting the need for culturally-sensitive approaches to promoting social engagement in these populations."

We observed gender differences in social participation patterns. Women participants were less likely to be in the working dominant cluster or the cluster of active social participation, which aligns with findings from China (Yuying & Jing, 2022) and Singapore (Ang, 2018) studies. One explanation could be the low labor participation among Taiwanese women born between 1920 and 1960, compared to many East Asian countries such as Japan and Korea (Yu). The poor labor market attachment could be due to historically unequal educational opportunities, marriage, and family duties (Chang, 2006). Moreover, our study participants grew up in a patriarchal era when women were expected to handle family responsibilities, while participating in activities in the public arena was considered men's responsibility (Xiao et al., 2021). Older Taiwanese women are more likely to be socialized into traditional gender roles like "kin-keeping" (Di, 1987) - maintaining family connections. While this expands women's networks within the extended family, it restricts engagement beyond the family circle. Interestingly, this contradicts patterns in Japan, where retired women maintain participation through hobbies or neighbor groups (Shiba et al., 2017). By contrast, Japanese men traditionally connect through work, becoming isolated in retirement (Takashima et al., 2020). The low participation in employment and social activities and the gap in educational attainment could largely explain the gender disparities in cognitive function observed in the Taiwanese older adult population. With advancing gender equality, the observed gaps in social participation and cognitive function may narrow. Research shows these differences disappear in more gender-equal countries (Van Tuyckom et al., 2013), while a Dutch study revealed a cohort effect where younger generations of women demonstrate higher social participation than their predecessors (Van Groenou & Deeg, 2010).

Lastly, we found that non-urban residents and those in the younger subgroup who had moved to more rural or more urban regions were more likely to belong to the cluster of active social participation than those in urban regions. Previous studies have reported mixed findings with regard to urban-rural differences in social participation. Some studies found that rural residents had lower social participation than their urban counterparts (Levasseur, Cohen, et al., 2015; Vogelsang, 2016). Some reasons for low social participation could be lower accessibility and proximity of social infrastructure (Levasseur et al., 2011, 2015a; Richard et al., 2013), low population density, and limited physical amenities (Neville et al., 2016). On the other hand, some studies reported that elderly residents in rural regions reported a higher level of bonding social capital (Norstrand & Xu, 2012) and stronger social ties (Levasseur, Cohen, et al., 2015). The phenomenon could be explained by the local norm of exchanging support among family members and the neighborhood in rural areas (Hofferth & Iceland, 1998). What we found in our analyses could be partially explained by the fact that Taiwan has a high-density population; in fact, Taiwan's rural population ranks higher than suburban densities in the United States (Thompson, 1984). Also, non-urban residents usually live closer to their family or relatives (Logan & Spitze, 1994). They move less frequently and are more likely to live in neighborhoods with long-time friends and neighbors. Therefore, most of the residents in the rural regions could still have access to social and physical infrastructure for socializing. Moreover, living in non-urban regions enables older people to continue working in farming or other traditional industries.

These trends may change in the future due to Taiwan's rapid rate of urbanization and outmigration from rural regions among younger people (Liao, 1988; Liu, 2019). The urbanization rate increased from 68.4 % in 1995 (near the time of the baseline survey year of this study) to 78.9 % in 2020. (Statista) Moreover, many counties and towns in Taiwan have been experiencing urban shrinkage since the end of the 20th century, with populations in some suburban regions and smaller cities decreasing over time (Hu, 2021). Along with rapid aging and low fertility (Lin et al., 2015), Taiwan is experiencing negative population growth (Hu, 2021), which may lead to difficulties in sustaining the social, economic, and cultural aspects of the suburban and rural systems. Therefore, the rural-urban difference in social participation that we found could disappear or even be reversed in the future. The Taiwanese government has emphasized regional revitalization as one of its priorities in 2020, promoting social participation as one of the five goals of the policy (National Development Council (Taiwan), 2017). The concept of regional revitalization was similarly adopted by Japan, and building an "active life town" system for middle-aged and elderly people to lead a healthy and active life is one of the main aspects in Japan's policy. (Yamauchi) Some of the regional revitalization cases in Taiwan have incorporated the active life town concept; for example, a project in Southern Taiwan converted an abandoned school in a rural region to a local community center for older residents to gather (Tsai et al., 2023). Nevertheless, Taiwan's region revitalization policy primarily focuses on encouraging the younger generation to stay or return to their hometowns to develop their careers. (Department of Information Services Executive Yuan) There is less discussion on how older people can participate in the initiative. Efforts on building a place to support a healthy and active life for both the young and older population could be a key to ensuring the success of the policy.

Our study has several strengths. First, it is among the first studies examining the life histories of social participation and work patterns in relation to cognition in middle-aged and older adults. By using optimal matching, we adequately account for social activities' ordering, duration, and timing. Second, we utilized a theory-based framework to categorize participation types and compare their cognitive impacts. Third, we clarified how sociodemographic factors shape participation patterns. Notably, we highlighted sex disparities and the advantages of rural social participation. These findings can advance knowledge on barriers and facilitators to social involvement, informing interventions to promote successful aging.

Several limitations of our study should be noted. First, we focused on individual determinants due to data constraints, not neighborhood or community factors like amenities and infrastructure facilitating participation. Incorporating family, social environment, and broader community context could provide a more comprehensive understanding of how older adults socialize and work. Second, most data were self-reported, resulting in potential information bias. Third, the study had a significant loss to follow-up. Even though we accounted for the lost to follow-up through censoring weights, bias due to differential drop-out might still remain. Last, 20–35 % of participants reported no social participation; while the rate was comparable to some Asian studies, we may have missed some informal interactions.

Our study provides some directions for future research. Future studies could consider further exploring the timing and time-varying social participation patterns to formally test the causal relationship between social engagement and cognitive function. Investigators could explore whether preventive interventions implemented earlier in life may be more effective in maintaining cognitive health than those initiated after cognitive decline has begun. Additionally, given our finding of declining social participation with aging across both subgroups, future research could elucidate the reasons behind this decline, identify risk factors for social disengagement, and discover potential promoters of sustained active social participation throughout later life. Based on our findings, interventions should consider cultural context and gender differences, as older women and men face different barriers and have heterogeneous needs regarding social engagement. Culturally adapted interventions that account for local customs, gender, family roles, and community-specific factors are more likely to be effective and equitable. Digital infrastructure and internet-based programs could be a solution for expanding participation opportunities, especially in areas with limited physical infrastructure (Ma & Sheng, 2025).

In conclusion, we examined lifecourse histories of social participation and working patterns in Taiwanese elders. We found clusters with multiple participation types and those dominated by working tended to be associated with higher cognition, supporting the value of social engagement and work involvement for healthy aging.

## CRediT authorship contribution statement

**Yu-Tien Hsu:** Writing – review & editing, Writing – original draft, Visualization, Validation, Supervision, Software, Resources, Project administration, Methodology, Investigation, Formal analysis, Data curation, Conceptualization. **Hanno Hoven:** Writing – review & editing, Visualization, Supervision, Software, Resources, Methodology, Investigation, Conceptualization. **Francine Grodstein:** Writing – review & editing, Validation, Supervision, Methodology, Investigation, Conceptualization. **Tzu-Hung Liu:** Writing – review & editing, Validation, Supervision, Resources, Project administration, Investigation, Data curation, Conceptualization. **Chia-Rui Chang:** Writing – review & editing, Validation, Methodology, Investigation, Conceptualization. **Yu-Lin Hsieh:** Writing – review & editing, Methodology, Investigation, Conceptualization. **Jarvis T. Chen:** Writing – review & editing, Visualization, Validation, Supervision, Methodology, Investigation, Formal analysis, Conceptualization. **Ichiro Kawachi:** Writing – review & editing, Writing – original draft, Visualization, Validation, Supervision, Resources, Project administration, Methodology, Investigation, Formal analysis, Data curation, Conceptualization.

## Consent statement

Our team did not obtain informed consent directly from the participants. However, the Taiwan Longitudinal Study on Aging research team (TLSA) provided a proper explanation of the study and informed consent for inclusion in the TLSA during participant recruitment. Participants who could read and write signed the written consent documents; those who could not read or write impressed their name stamps or fingerprints with the assistance of family members.

## Ethical statement

This study, entitled “Social Participation Trajectories in Late Life and Cognitive Functioning – A Sequence Analysis Based on Taiwan Longitudinal Study on Aging,” was approved by the Harvard T.H. Chan School of Public Health's IRB committee (IRB20-0969).

## Funding information

The authors have no funding information to disclose.

## Declaration of competing interest

The authors declare that they have no known competing financial interests or personal relationships that could have appeared to influence the work reported in this paper.

## Data Availability

Data will be made available on request.
