# Supplementary material for: Social participation trajectories in late life and cognitive functioning – A sequence analysis based on Taiwan Longitudinal Study on aging
Source: SSM Popul Health. 2025 May 28;31:101821. doi: 10.1016/j.ssmph.2025.101821 (PMC12182371; doi:10.1016/j.ssmph.2025.101821)
Supplement: Multimedia component 1 [file mmc1.docx]

**SUPPLEMENTARY MATERIALS**

**Supplementary Table 1. Participants with full information on social participation and working at each wave in two subgroups**

|  | **1996** | **1999** | **2003** | **2007** | **2011** |
| --- | --- | --- | --- | --- | --- |
| **Middle-Aged Subgroup (Age 50-64 in 1996)** | | | | | |
| Number of participants | 1,900 | 1,637 | 1,587 | 1,472 | 1,305 |
| Number of people lost to follow-up (%) |  | 263 (13.8%) | 50  (3.1%) | 115 (7.2%) | 167 (11.3%) |
| **Older Subgroup (Age 65+ in 1996)** | | | | | |
| Number of participants | 2,500 | 2,090 | 1,658 | 1,232 | 812 |
| Number of people lost to follow-up (%) |  | 410 (16.4%) | 332 (15.9%) | 426 (25.7%) | 420 (34.1%) |

**Supplementary Table 2. Cluster quality assessment**

| **Number of clusters** | **Younger Subgroup (Age 50-65 in 1996)** | | | **Older subgroup (Age 65+ in 1996)** | | |
| --- | --- | --- | --- | --- | --- | --- |
|  | Average Silhouette Width (ASW) | Hubert’s Gamma (HG) | Point Biserial Correlation (PBC) | Average Silhouette Width (ASW) | Hubert’s Gamma (HG) | Point Biserial Correlation (PBC) |
| 5 | 0.131 | 0.520 | 0.401 | 0.198 | 0.513 | 0.371 |
| 6 | 0.105 | 0.539 | 0.373 | 0.190 | 0.542 | 0.359 |
| 7 | 0.109 | 0.568 | 0.381 | 0.188 | 0.567 | 0.363 |
| 8 | 0.091 | 0.570 | 0.351 | 0.198 | 0.595 | 0.370 |

**Supplementary Table 3. Summary statistics of social participation and working histories**

|  | **Younger Subgroup (Age 50-65 in 1996)** | **Older Subgroup (Age 65+ in 1996)** |
| --- | --- | --- |
| **Mean cumulative state duration (waves)** |  |  |
| State A: helping, working, and socializing | 0.32 | 0.09 |
| State B: helping and working | 0.23 | 0.04 |
| State C: helping and socializing | 0.49 | 0.33 |
| State D: working and socializing | 0.44 | 0.15 |
| State E: socializing only | 0.48 | 0.70 |
| State F: working only | 0.64 | 0.15 |
| State G: helping only | 0.55 | 0.35 |
| State H: did not involve in any of the social participation types | 1.00 | 1.51 |
| **Mean number of state transition an individual made** | 1.95 | 1.18 |
| **Shannon’s Entropy (SD)^*^** | 0.39 (0.20) | 0.25 (0.21) |

^*^ Shannon’s entropy measures variability associated with random variables. The formula for calculating Shannon’s entropy is as follows (Formula 2):

$H=\sum_{i=1}^{s} -(P_{i}-lnP_{i})$ (Formula 2)

where, H indicates Shannon’s entropy, $P_{i}$ refers to the entire population made up of individuals with each type of sequence of social participation and working history i, ln($P_{i}$) is the natural log of above, and S is the total number of types of social participation and working sequences. Shannon’s entropy captures how concentrated or dispersed the distribution of social participation and working histories is for a given population. A higher value of a Shannon’s entropy indicates greater between-person diversity of social participation and working history.

**Supplementary Table 4. Participants with full information on social participation and working at each wave in two subgroups**

|  | **1996** | **1999** | **2003** | **2007** | **2011** |
| --- | --- | --- | --- | --- | --- |
| **Middle-Aged Subgroup (Age 50-64 in 1996)** | | | | | |
| Number of participants | 1,900 | 1,637 | 1,587 | 1,472 | 1,305 |
| Number of people lost to follow-up (%) |  | 263 (13.8%) | 50  (3.1%) | 115 (7.2%) | 167 (11.3%) |
| **Older Subgroup (Age 65+ in 1996)** | | | | | |
| Number of participants | 2,500 | 2,090 | 1,658 | 1,232 | 812 |
| Number of people lost to follow-up (%) |  | 410 (16.4%) | 332 (15.9%) | 426 (25.7%) | 420 (34.1%) |

**Supplementary Table 5. Missingness (%) in variables**

| **Variable** | **Total Sample** | **Older Subgroup (Age 65+ in 1996)** | **Middle-aged subgroup (Age 50-64 in 1996)** |
| --- | --- | --- | --- |
| **No. Participants** | 4,400 | 2,500 (100%) | 1,900 (100%) |
| **Gender** | 0 (0%) | 0 (0%) | 0 (0%) |
| **Age** | 0 (0%) | 0 (0%) | 0 (0%) |
| **Education** | 0 (0%) | 0 (0%) | 0 (0%) |
| **Ethnicity** | 9 (0.2%) | 8 (0.3%) | 1 (0.05%) |
| **Marital Status** | 228 (5.2%) | 39 (1.6%) | 189 (10.0%) |
| **Urbanicity of Residence** | 39 (0.9%) | 22 (1.0%) | 17 (0.9%) |
| **Medical Condition** | | |  |
| **Hypertension** | 0 (0%) | 0 (0%) | 0 (0%) |
| **Diabetes** | 0 (0%) | 0 (0%) | 0 (0%) |
| **Cancer** | 0 (0%) | 0 (0%) | 0 (0%) |
| **Baseline mean CESD Score (SD)** | 169 (3.8%) | 45 (1.8%) | 124 (6.5%) |
| **Cognitive function score in 2015** | 0 (0%) | 0 (0%) | 0 (0%) |
| **Social Participation State** | | | |
| **1996** | 2 (0.08%) | 2 (0.1%) | 0 (0 %) |
| **1999** | 16 (0.6%) | 8 (0.4%) | 8 (0.5%) |
| **2003** | 1 (0.04%) | 1 (0.06%) | 0 (0%) |
| **2007** | 2 (0.08%) | 0 (0%) | 2 (0.1%) |
| **2011** | 0 (0%) | 0 (0%) | 0 (0%) |

**Supplementary Table 6. Multinomial logistic regression predicting social participation cluster membership (reference group: low social participation cluster, middle-aged subgroup (age 50-64 in 1996) (i.e., Cluster 2)). (N = 1,900)**

|  | **RRR (95% C.I., p-value)** | | | | |
| --- | --- | --- | --- | --- | --- |
|  | **Cluster 1** | **Cluster 3** | **Cluster 4** | **Cluster 5** | **Cluster 6** |
| **Age (ref: 50-54 year-old)** | | | | | |
| 55-59 year-old | 0.72 (95% C.I. = 0.46–1.13, p = 0.097) | **0.33 (95% C.I. = 0.22, 0.51, p<0.001)** | **0.44 (95% C.I. = 0.29–0.66, p<0.001)** | 0.93 (95% C.I. = 0.56–1.55, p = 0.786) | 0.69 (95% C.I. = 0.44–1.10, p = 0.116) |
| 60-64 year-old | 0.89 (95% C.I. = 0.56–1.42, p = 0.393) | **0.29 (95% C.I. = 0.18, 0.47, p<0.001)** | **0.29 (95% C.I. = 0.18–0.48, p<0.001)** | 1.57 (95% C.I. = 0.94–2.61, p = 0.083) | 0.92 (95% C.I. = 0.57–1.49, p = 0.741) |
| **Education level (ref: no education)** | | | | | |
| Elementary school | 1.49 (95% C.I. = 0.93–2.39, p = 0.097) | 1.04 (95% C.I. = 0.62, 1.76, p = 0.871) | 0.92 (95% C.I. = 0.56–1.52, p = 0.746) | 1.04 (95% C.I. = 0.65–1.68, p = 0.384) | 0.81 (95% C.I. = 0.51–­1.29, p = 0.384) |
| Middle/High school | 1.31 (95% C.I. = 0.70–2.44, p = 0.393) | 0.78 (95% C.I. = 0.41, 1.50, p = 0.466) | 0.86 (95% C.I. = 0.46–1.58, p = 0.619) | 1.09 (95% C.I. = 0.57–2.07, p = 0.437) | 0.79 (95% C.I. = 0.43–1.44, p = 0.437) |
| College or above | 1.81 (95% C.I. = 0.77–4.28, p = 0.177) | 1.34 (95% C.I. = 0.58, 3.09, p = 0.496) | 0.48 (95% C.I. = 0.19–1.18, p = 0.110) | 0.88 (95% C.I. = 0.31–2.46, p = 0.971) | 1.02 (95% C.I. = 0.42–2.48, p = 0.971) |
| **Marital Status (ref: married participants)** | | | | | |
| Widowed / Divorced | 0.85 (95% C.I. = 0.57–1.28, p = 0.448) | 1.04 (95% C.I. = 0.67, 1.60, p = 0.866) | 0.76 (95% C.I. = 0.49–1.18, p = 0.219) | 1.45 (95% C.I. = 0.97–2.18, p = 0.890) | 1.03 (95% C.I. = 0.68 – 1.55, p = 0.890) |
| **Urbanicity (ref: urban residents)** | | | | | |
| Township | 1.08 (95% C.I. = 0.48–2.41, p = 0.853) | 1.37 (95% C.I. = 0.68–2.79, p = 0.385) | **2.50 (95% C.I. = 1.24–5.07, p = 0.011)** | **2.43 (95% C.I. = 1.08–5.47, p = 0.031)** | 0.59 (95% C.I. = 0.26–1.33, p = 0.203) |
| Rural region | **2.84 (95% C.I. = 1.59–5.08, p<0.001)** | 1.40 (95% C.I. = 0.78–2.51, p = 0.268) | **4.06 (95% C.I. = 2.33–7.09, p<0.001)** | **3.86 (95% C.I. = 1.98–7.50, p<0.001)** | **0.49 (95% C.I. = 0.24–0.99, p = 0.045)** |
| Moving to more rural regions | **1.85 (95% C.I. = 1.07**–**3.18, p = 0.027)** | 0.96 (95% C.I. = 0.56–1.66, p = 0.891) | **1.76 (95% C.I. = 1.03–3.03, p = 0.041)** | **2.99 (95% C.I. = 1.61–5.56, p<0.001)** | 0.89 (95% C.I. = 0.52–1.52, p = 0.665) |
| Moving to more urban regions | **2.41 (95% C.I. = 1.49**–**3.90, p<0.001)** | 1.40 (95% C.I. = 0.87, 2.26, p = 0.164) | **2.87 (95% C.I. = 1.77–4.66, p<0.001)** | **4.48 (95% C.I. = 2.59–7.74, p<0.001)** | 1.03 (95% C.I. = 0.64–1.64, p = 0.910) |
| **Female (ref. male)** | 1.58 (95% C.I. = 0.86–2.90, p = 0.142) | **0.29 (95% C.I. =** **0.16–0.54, p<0.001)** | **0.11 (95% C.I. = 0.06–0.20, p<0.001)** | 1.12 (95% C.I. = 0.59–2.11, p = 0.733) | 1.66 (95% C.I. = 0.87–3.16, p = 0.123) |
| **Medical Condition** | | | | | |
| Hypertension | 0.88 (95% C.I. = 0.61–1.28, p = 0.500) | **0.65 (95% C.I. =** **0.45–0.94, p = 0.021)** | 0.75 (95% C.I. = 0.53–1.07, p = 0.115) | 0.91 (95% C.I. = 0.61–1.35, p = 0.644) | 0.72 (95% C.I. = 0.49–1.05, p = 0.091) |
| Diabetes | 0.88 (95% C.I. = 0.59–1.31, p = 0.527) | 0.74 (95% C.I. = 0.49–1.12, p = 0.148) | 0.72 (95% C.I. = 0.49–1.07, p = 0.105) | 0.78 (95% C.I. = 0.51­–1.19, p = 0.250) | 0.84 (95% C.I. = 0.56–1.27, p = 0.412) |
| Cancer | 1.00 (95% C.I. = 0.55–1.82, p = 0.987) | **0.31 (95% C.I. = 0.14–0.73, p = 0.007)** | **0.42 (95% C.I. = 0.20–0.89, p = 0.023)** | 1.04 (95% C.I. = 0.55 –1.97, p = 0.888) | 0.64 (95% C.I. = 0.33–1.22, p = 0.176) |
| CESD Score | 0.97 (95% C.I. = 0.93–1.02, p = 0.236) | 0.98 (95% C.I. = 0.94–1.03, p = 0.405) | **0.94 (95% C.I. = 0.90–0.99, p = 0.012)** | 0.97 (95% C.I. = 0.93 –1.01, p = 0.175) | 1.00 (95% C.I. = 0.96–1.04, p = 0.957) |
| **Health Behaviors** | | | | | |
| Smoking | 0.59 (95% C.I. = 0.32–2.82, p = 0.096) | 0.65 (95% C.I. = 0.37–1.17, p = 0.150) | **0.39 (95% C.I. = 0.23–0.67, p = 0.001)** | **0.37 (95% C.I. = 0.19–0.72, p = 0.003)** | 1.07 (95% C.I. = 0.56–2.06, p = 0.820) |
| Alcohol drinking | **1.81 (95% C.I. =** **1.16**–**2.82, p = 0.009)** | 0.87 (95% C.I. = 0.55–1.39, p = 0.570) | 0.84 (95% C.I. = 0.53–1.32, p = 0.446) | **2.22 (95% C.I. = 1.38 – 3.58, p = 0.001)** | 0.98 (95% C.I. = 0.62–1.55, p = 0.920) |
| Betel nut use | 0.76 (95% C.I. = 0.43–1.37, p = 0.362) | 0.91 (95% C.I. = 0.54–1.52, p = 0.715) | 1.48 (95% C.I. = 0.94–2.33, p = 0.093) | 1.15 (95% C.I. = 0.64 – 2.05, p = 0.632) | 0.57 (95% C.I. = 0.28–1.13, p = 0.108) |
| Regular exercise | **2.62 (95% C.I. =** **1.39–4.97; 0.003)** | 0.87 (95% C.I. = 0.53–1.41, p = 0.568) | 0.95 (95% C.I. = 0.59–1.51, p = 0.823) | **2.34 (95% C.I. = 1.22–4.46, p = 0.010)** | 1.13 (95% C.I. = 0.67–1.91, p = 0.643) |

Abbreviation: RRR, Relative Risk Ratio. Statistically significant estimates in bold.

**Supplementary Table 7. Multinomial logistic regression predicting social participation cluster membership (reference group: low social participation cluster, older subgroup (age 65+ in 1996) (i.e., Cluster 3)). (N = 2,500)**

|  | **RRR (95% C.I., p-value)** | | | | |
| --- | --- | --- | --- | --- | --- |
|  | **Cluster 1** | **Cluster 2** | **Cluster 4** | **Cluster 5** | **Cluster 6** |
| **Age (ref: 65-69 year-old)** | | | | | |
| 70-74 year-old | 1.01 (95% C.I. = 0.70–1.45, p = 0.958) | 0.93 (95% C.I. = 0.62–1.39, p = 0.730) | 0.79 (95% C.I. = 0.54–1.51, p = 0.218) | 0.80 (95% C.I. = 0.51–1.25, p = 0.321) | 0.83 (95% C.I. = 0.55–1.27, p = 0.396) |
| 75-79 year-old | 1.10 (95% C.I. = 0.77–1.57, p = 0.595) | 0.94 (95% C.I. = 0.63–1.40, p = 0.756) | 0.82 (95% C.I. = 0.57–1.19, p = 0.304) | 0.87 (95% C.I. = 0.56–1.34, p = 0.517) | 0.89 (95% C.I. = 0.59–1.36, p = 0.598) |
| 80 year-old or above | **1.97 (95% C.I. = 1.11–3.48, p = 0.020)** | 1.64 (95% C.I. = 0.84–3.17, p = 0.145) | **2.22 (95% C.I. = 1.27–3.87, p = 0.005)** | 1.52 (95% C.I. = 0.74–3.14, p = 0.258) | **2.40 (95% C.I. = 1.29–4.47, p = 0.006)** |
| **Education level (ref: no education)** | | | | | |
| Elementary school | 1.09 (95% C.I. = 0.77–1.53, p = 0.632) | 1.06 (95% C.I. = 0.70–1.60, p = 0.778) | 0.94 (95% C.I. = 0.66–1.35, p = 0.751) | **1.74 (95% C.I. = 1.08–2.81, p = 0.024)** | 1.33 (95% C.I. = 0.89­ – 2.00, p = 0.168) |
| Middle/High school | 1.05 (95% C.I. = 0.66–1.68, p = 0.830) | 1.12 (95% C.I. = 0.67–1.88, p = 0.663) | 0.64 (95% C.I. = 0.39–1.05, p = 0.079) | **3.46 (95% C.I. = 1.97–6.08, p<0.001)** | 0.91 (95% C.I. = 0.52 – 1.59, p = 0.741) |
| College or above | 1.16 (95% C.I. = 0.57–2.36, p = 0.680) | 1.31 (95% C.I. = 0.65–2.62, p = 0.454) | 1.02 (95% C.I. = 0.52–2.02, p = 0.950) | **2.91 (95% C.I. = 1.30–6.48, p<0.001)** | 0.91 (95% C.I. = 0.40 – 2.10, p = 0.828) |
| **Marital Status (ref: married participants)** | | | | | |
| Widowed / Divorced | 0.92 (95% C.I. = 0.67–1.28, p = 0.638) | **0.48 (95% C.I. = 0.34–0.69, p < 0.001)** | **0.65 (95% C.I. = 0.47–0.91, p = 0.011)** | 0.67 (95% C.I. = 0.45–1.00, p = 0.051) | **0.55 (95% C.I. = 0.38 – 0.79, p = 0.001)** |
| **Urbanicity (ref: urban residents)** | | | | | |
| Township | **2.12 (95% C.I. = 1.20–3.76, p = 0.010)** | 1.47 (95% C.I. = 0.80–2.68, p = 0.213) | **1.84 (95% C.I. = 1.08–3.11, p = 0.023)** | **2.61 (95% C.I. = 1.35–5.05, p = 0.004)** | **3.97 (95% C.I. = 2.10–7.51, p<0.001)** |
| Rural region | **4.30 (95% C.I. = 2.66–6.95, p<0.001)** | 1.54 (95% C.I. = 0.89–2.67, p = 0.121) | **2.04 (95% C.I. = 1.27–3.28, p = 0.003)** | **4.30 (95% C.I. = 2.45–7.57, p<0.001)** | **11.29 (95% C.I. = 6.65–19.19, p<0.001)** |
| Moving to more rural regions | **1.79 (95% C.I. = 1.17–2.73, p = 0.007)** | 0.64 (95% C.I. = 0.38–1.06, p = 0.085) | **0.57 (95% C.I. = 0.36–0.93, p = 0.023)** | 1.63 (95% C.I. = 0.98–2.74, p = 0.062) | **1.83 (95% C.I. = 1.05–3.20, p = 0.034)** |
| Moving to more urban regions | **1.87 (95% C.I. = 1.29–2.71, p = 0.001)** | 0.91 (95% C.I. = 0.61–1.36, p = 0.644) | 0.67 (95% C.I. = 0.45–1.01, p = 0.053) | 1.22 (95% C.I. = 0.76–1.97, p = 0.408) | **2.32 (95% C.I. = 1.43 – 3.74, p<0.001)** |
| **Female** | 0.99 (95% C.I. = 0.62–1.58, p = 0.956) | **0.35 (95% C.I. = 0.21–0.60, p<0.001)** | **0.39 (95% C.I. = 0.24–0.64, p<0.001)** | 0.61 (95% C.I. = 0.34–1.09, p = 0.094) | **0.19 (95% C.I. = 0.11–0.32, p<0.001)** |
| **Baseline Medical Condition** | | | | | |
| Hypertension | 0.87 (95% C.I. = 0.62–1.58, p = 0.372) | **0.65 (95% C.I. = 0.46–0.90, p = 0.010)** | **0.39 (95% C.I. = 0.29–0.53, p<0.001)** | **0.67 (95% C.I. = 0.46–0.97, p = 0.032)** | **0.52 (95% C.I. = 0.37–0.73, p<0.001)** |
| Diabetes | 0.83 (95% C.I. = 0.60–1.31, p = 0.272) | 1.22 (95% C.I. = 0.85–1.75, p = 0.276) | 0.83 (95% C.I. = 0.59–1.19, p = 0.312) | 1.17 (95% C.I. = 0.79­–1.73, p = 0.445) | 0.84 (95% C.I. = 0.56–1.25, p = 0.390) |
| Cancer | 1.28 (95% C.I. = 0.74–2.20, p = 0.373) | 1.29 (95% C.I. = 0.72–2.31, p = 0.394) | 0.74 (95% C.I. = 0.39–1.41, p = 0.361) | 1.71 (95% C.I. = 0.94 –3.11, p = 0.080) | 1.24 (95% C.I. = 0.65–2.33, p = 0.513) |
| CESD Score | 1.01 (95% C.I. = 0.98–1.05, p = 0.360) | 1.01 (95% C.I. = 0.98–1.05, p = 0.537) | 1.02 (95% C.I. = 0.98–1.05, p = 0.336) | 0.99 (95% C.I. = 0.95 –1.03, p = 0.597) | 0.98 (95% C.I. = 0.94 – 1.01, p = 0.192) |
| **Health Behaviors** | | | | | |
| Smoking | 1.33 (95% C.I. = 0.84–2.08, p = 0.220) | 1.01 (95% C.I. = 0.63–1.63, p = 0.952) | 1.25 (95% C.I. = 0.79–1.97, p = 0.338) | 1.11 (95% C.I. = 0.65–1.88, p = 0.698) | 1.03 (95% C.I. = 0.63–1.70, p = 0.901) |
| Alcohol drinking | 0.93 (95% C.I. = 0.64–1.33, p = 0.677) | 0.96 (95% C.I. = 0.64–1.63, p = 0.826) | **0.58 (95% C.I. = 0.40–0.84, p = 0.004)** | 0.75 (95% C.I. = 0.48 – 1.16, p = 0.194) | **0.50 (95% C.I. = 0.33–0.76, p<0.001)** |
| Betel nut use | 1.16 (95% C.I. = 0.68–1.96, p = 0.592) | 0.52 (95% C.I. = 0.26–1.04, p = 0.064) | 0.80 (95% C.I. = 0.45–1.43, p = 0.453) | 0.98 (95% C.I. = 0.50 – 1.90, p = 0.945) | **1.93 (95% C.I. = 1.14–3.26, p = 0.015)** |
| Regular exercise | 1.33 (95% C.I. = 0.84–2.10, p = 0.231) | **0.63 (95% C.I. = 0.40–0.99, p = 0.048)** | **0.30 (95% C.I. = 0.21–0.45, p<0.001)** | 1.08 (95% C.I. = 0.61–2.22, p = 0.789) | **0.48 (95% C.I. = 0.31–0.76, p = 0.001)** |

Abbreviation: RRR, Relative Risk Ratio. Statistically significant estimates in bold

**Supplementary Table 8. Subgroup Analysis by Age for the Association between social participation clustering and score on the Short Portable Mental Status Questionnaire using OLS regression and censoring weights, Middle-Aged Group (N = 1,900)^*^**

| **Cognitive Score (in 2015)** | | | | | | |
| --- | --- | --- | --- | --- | --- | --- |
| **Middle-aged subgroup (age 50-64 in 1996). (N = 1,900)** | | | | | | |
|  | **Age 50-54** | | **Age 54-59** | | **Age 60-64** | |
| **Cluster** | **Mean Difference** | **95% C.I.** | **Mean Difference** | **95% C.I.** | **Mean Difference** | **95% C.I.** |
| Cluster 2: Low social participation (Ref)  Cluster 1: Socializing & helping  Cluster 3: Working  Cluster 4: Active in multiple states  Cluster 5: Socializing  Cluster 6: Helping | –  **0.57**  0.33  0.33  0.31  -0.15 | –  **(0.10, 1.04)***  (-0.14, 0.80)  (-0.17, 0.85)  (-0.28, 0.89)  (-0.82, 0.51) | –  0.55  **0.04**  **0.02**  0.51  0.81 | –  (-0.34, 1.44)  (-0.26, 1.23)  (-0.05, 1.82)  (-1.18, 0.63)  (-0.83, 0.98) | –  0.78  0.95  1.35  -0.09  1.01 | –  (-0.41, 1.97)  (-0.52, 2.42)  (0.25, 2.44)  (-1.35, 1.16)  (-0.09, 2.12) |

^*^Adjusted for marital status, educational attainment, urbanicity of residence, medical diagnosis (cancer, hypertension, diabetes), baseline CESD score, health behaviors (smoking, drinking, betel nut use, regular exercise), number of grandchildren. Statistically significant estimates in bold.

**Supplementary Table 9. Subgroup Analysis by Gender for the Association between social participation clustering and score on the Short Portable Mental Status Questionnaire using OLS regression and censoring weights, Middle-Aged Group (N = 1,900)^*^**

| **Cognitive Score (in 2015)** | | | | |
| --- | --- | --- | --- | --- |
| **Middle-aged subgroup (age 50-64 in 1996). (N = 1,900)** | | | | |
|  | **Female** | | **Male** | |
| **Cluster** | **Mean Difference** | **95% C.I.** | **Mean Difference** | **95% C.I.** |
| Cluster 2: Low social participation (Ref)  Cluster 1: Socializing & helping  Cluster 3: Working  Cluster 4: Active in multiple states  Cluster 5: Socializing  Cluster 6: Helping | –  **0.97**  **0.79**  **1.12**  0.33  0.51 | –  **(0.40, 1.54)****  **(0.19, 1.39)***  **(0.31, 1.94)****  (-0.35, 1.00)  (-0.10, 1.11) | –  -0.09  0.18  0.28  -0.96  -0.12 | –  (-0.91, 0.74)  (-0.42, 0.78)  (-0.29, 0.85)  (-2.21, 0.12)  (-1.08, 0.85) |

^*^Adjusted for marital status, educational attainment, urbanicity of residence, medical diagnosis (cancer, hypertension, diabetes), baseline CESD score, health behaviors (smoking, drinking, betel nut use, regular exercise), number of grandchildren. Statistically significant estimates in bold.

**Supplementary Table 10. Subgroup Analysis by Urbanicity for the Association between social participation clustering and score on the Short Portable Mental Status Questionnaire using OLS regression and censoring weights, Middle-Aged Group (N = 1,900)^*^**

| **Cognitive Score (in 2015)** | | | | | | |
| --- | --- | --- | --- | --- | --- | --- |
| **Middle-aged subgroup (age 50-64 in 1996). (N = 1,900)** | | | | | | |
|  | **Urban Region** | | **Township** | | **Rural Regions** | |
| **Cluster** | **Mean Difference** | **95% C.I.** | **Mean Difference** | **95% C.I.** | **Mean Difference** | **95% C.I.** |
| Cluster 2: Low social participation (Ref)  Cluster 1: Socializing & helping  Cluster 3: Working  Cluster 4: Active in multiple states  Cluster 5: Socializing  Cluster 6: Helping | –  0.52  0.37  0.41  -0.24  -0.07 | –  (-0.19, 1.23)  (-0.34, 1.07)  (-0.43, 1.26)  (-1.48, 1.01)  (-0.87, 0.72) | –  0.34  1.10  0.90  0.56  0.29 | –  (-1.02, 1.69)  (-0.04, 2.25)  (-0.17, 1.96)  (-0.50, 1.62)  (-1.09, 1.67) | –  0.25  0.01  0.26  **-1.64**  0.31 | –  (-0.78, 1.28)  (-1.27, 1.29)  (-0.90, 1.42)  **(-3.27, -0.01**)  (-0.96, 1.58) |

^*^Adjusted for marital status, educational attainment, urbanicity of residence, medical diagnosis (cancer, hypertension, diabetes), baseline CESD score, health behaviors (smoking, drinking, betel nut use, regular exercise), number of grandchildren. Statistically significant estimates in bold.

**Supplementary Table 11. Subgroup Analysis by Age for the Association between social participation clustering and score on the Short Portable Mental Status Questionnaire using OLS regression and censoring weights, Older Group (N = 2,500)^*^**

| **Cognitive Score (in 2015)** | | | | | | |
| --- | --- | --- | --- | --- | --- | --- |
| **Older subgroup (age 65+ in 1996). (N = 2,500)** | | | | | | |
|  | **Age 65-69** | | **Age 70-74** | | **Above 75** | |
| **Cluster** | **Mean Difference** | **95% C.I.** | **Mean Difference** | **95% C.I.** | **Mean Difference** | **95% C.I.** |
| Cluster 3: Low social participation (Ref)  Cluster 1: Socializing  Cluster 2: Helping  Cluster 4: Working & low social participation  Cluster 5: Helping & socializing  Cluster 6: Active in multiple states | –  **-0.36**  -0.53  0.72  **1.79**  -0.50 | –  **(-1.96, 1.24)**  (-2.47, 1.40)  (-0.45, 1.89)  **(0.54, 3.04)**  (-2.36, 1.36) | –  **2.75**  **2.47**  **3.69**  **2.65**  **6.39** | –  **(1.31, 4.19)*****  **(0.10, 4.85)***  **(0.43, 6.96)***  **(0.13, 5.16)***  **(4.21, 8.56)***** | –  0.15  1.40  3.03  3.13  **3.96** | –  (-1.77, 2.07)  (-1.93, 4.74)  (-0.11, 6.17)  (-0.24, 6.50)  **(0.33, 7.58)*** |

^*^Adjusted for marital status, educational attainment, urbanicity of residence, medical diagnosis (cancer, hypertension, diabetes), baseline CESD score, health behaviors (smoking, drinking, betel nut use, regular exercise), number of grandchildren. Statistically significant estimates in bold.

**Supplementary Table 12. Subgroup Analysis by Sex for the Association between social participation clustering and score on the Short Portable Mental Status Questionnaire using OLS regression and censoring weights, Older Group (N = 2,500)^*^**

| **Cognitive Score (in 2015)** | | | | |
| --- | --- | --- | --- | --- |
| **Older subgroup (age 65+ in 1996). (N = 2,500)** | | | | |
|  | **Female** | | **Male** | |
| **Cluster** | **Mean Difference** | **95% C.I.** | **Mean Difference** | **95% C.I.** |
| Cluster 3: Low social participation (Ref)  Cluster 1: Socializing  Cluster 2: Helping  Cluster 4: Working & low social participation  Cluster 5: Helping & socializing  Cluster 6: Active in multiple states | –  **1.06**  **1.10**  **1.48**  0.33  0.56 | –  **(0.47, 1.66)*****  **(0.49, 1.71)*****  **(0.63, 2.32)****  (-0.38, 1.04)  (-0.05, 1.16) | –  0.85  -0.28  **2.55**  1.27  0.36 | –  (-0.91, 2.60)  (-2.09, 1.54)  **(0.90, 4.19)****  (-0.03, 2.56)  (-1.32, 2.05) |

^*^Adjusted for marital status, educational attainment, urbanicity of residence, medical diagnosis (cancer, hypertension, diabetes), baseline CESD score, health behaviors (smoking, drinking, betel nut use, regular exercise), number of grandchildren. Statistically significant estimates in bold.

**Supplementary Table 13. Subgroup Analysis by Urbanicity for the Association between social participation clustering and score on the Short Portable Mental Status Questionnaire using OLS regression and censoring weights, Older Group (N = 2,500)^*^**

| **Cognitive Score (in 2015)** | | | | | | |
| --- | --- | --- | --- | --- | --- | --- |
| **Older subgroup (age 65+ in 1996). (N = 2,500)** | | | | | | |
|  | **Urban Region** | | **Township** | | **Rural Regions** | |
| **Cluster** | **Mean Difference** | **95% C.I.** | **Mean Difference** | **95% C.I.** | **Mean Difference** | **95% C.I.** |
| Cluster 3: Low social participation (Ref)  Cluster 1: Socializing  Cluster 2: Helping  Cluster 4: Working & low social participation  Cluster 5: Helping & socializing  Cluster 6: Active in multiple states | –  -0.41  **3.41**  **3.25**  **3.44**  **2.48** | –  (-2.71, 1.90)  **(2.03, 4.80)*****  **(1.19, 5.31)****  **(2.02, 4.87)*****  **(0.93, 4.03)**** | –  **-3.43**  1.49  -0.26  -0.95  1.60 | –  **(-6.10, -0.76)***  (-1.88, 4.87)  (-3.64, 3.13)  (-3.55, 1.65)  (-1.05, 4.24) | –  **2.67**  -1.60  0.79  **3.41**  -2.13 | –  **(0.84, 4.50)****  (-3.87, 0.67)  (-0.96, 2.54)  **(0.69, 6.13)***  (-4.68, 0.41) |

^*^Adjusted for marital status, educational attainment, urbanicity of residence, medical diagnosis (cancer, hypertension, diabetes), baseline CESD score, health behaviors (smoking, drinking, betel nut use, regular exercise), number of grandchildren. Statistically significant estimates in bold.

**Supplementary Table 14. State**^†^ **transition probabilities in two consecutive waves, aggregating data from all waves, younger subgroup (age 50-65 in 1996).^,^ (N=1,900)**

| **State** | **A** | **B** | **C** | **D** | **E** | **F** | **G** | **H** |
| --- | --- | --- | --- | --- | --- | --- | --- | --- |
| **A** | 0.30 | 0.08 | 0.14 | 0.19 | 0.08 | 0.09 | 0.05 | 0.06 |
| **B** | 0.14 | 0.12 | 0.07 | 0.14 | 0.05 | 0.22 | 0.11 | 0.15 |
| **C** | 0.03 | 0.01 | 0.42 | 0.03 | 0.18 | 0.01 | 0.15 | 0.17 |
| **D** | 0.13 | 0.03 | 0.07 | 0.33 | 0.13 | 0.17 | 0.04 | 0.09 |
| **E** | 0.02 | 0.01 | 0.18 | 0.03 | 0.4 | 0.02 | 0.09 | 0.25 |
| **F** | 0.06 | 0.1 | 0.04 | 0.15 | 0.04 | 0.36 | 0.09 | 0.17 |
| **G** | 0.02 | 0.02 | 0.17 | 0.01 | 0.10 | 0.02 | 0.36 | 0.32 |
| **H** | 0.01 | 0.01 | 0.07 | 0.01 | 0.14 | 0.04 | 0.16 | 0.57 |

^†^ Social participation state A: helping, working, and socializing; State B: helping and working; State C: helping and socializing; State D: working and socializing; State E: socializing only; State F: working only; State G: helping only; State H: did not involve in any of the social participation types.

**Supplementary Table 15. State^‡^ transition probabilities in two consecutive waves, aggregating data from all waves, older subgroup (age 65+ in 1996). (N=2,500)**

| **State** | **A** | **B** | **C** | **D** | **E** | **F** | **G** | **H** |
| --- | --- | --- | --- | --- | --- | --- | --- | --- |
| **A** | 0.22 | 0.04 | 0.12 | 0.18 | 0.19 | 0.14 | 0.04 | 0.07 |
| **B** | 0.10 | 0.13 | 0.06 | 0.10 | 0.06 | 0.20 | 0.15 | 0.20 |
| **C** | 0.01 | 0.00 | 0.27 | 0.01 | 0.34 | 0.00 | 0.10 | 0.26 |
| **D** | 0.09 | 0.01 | 0.07 | 0.31 | 0.25 | 0.11 | 0.03 | 0.15 |
| **E** | 0.00 | 0.00 | 0.10 | 0.01 | 0.45 | 0.00 | 0.03 | 0.40 |
| **F** | 0.05 | 0.03 | 0.04 | 0.11 | 0.11 | 0.28 | 0.06 | 0.31 |
| **G** | 0.00 | 0.01 | 0.09 | 0.00 | 0.10 | 0.01 | 0.26 | 0.53 |
| **H** | 0.00 | 0.00 | 0.03 | 0.00 | 0.12 | 0.01 | 0.09 | 0.74 |

^‡^ Social participation state A: helping, working, and socializing; State B: helping and working; State C: helping and socializing; State D: working and socializing; State E: socializing only; State F: working only; State G: helping only; State H: did not involve in any of the social participation types.
