# Supplementary material for: Social participation trajectories in late life and cognitive functioning – A sequence analysis based on Taiwan Longitudinal Study on aging
Source: SSM Popul Health. 2025 May 28;31:101821. doi: 10.1016/j.ssmph.2025.101821 (PMC12182371; doi:10.1016/j.ssmph.2025.101821)
Supplement: Multimedia component 2 [file mmc2.docx]

**Appendix A. Categorizations of social participation and working**

A participant’s working status was defined as whether one was working or helping with his/her family’s business or farming (yes/no). We conceptualized social participation based on the framework proposed by Levasseur et al, which categorized social activities into six levels.^15^ Levels 1-3 include activities that one performs alone or with limited interactions with others. Level 4 activities are those performed with others with a common goal, for example, spending time socializing with family or friends or participating in hobby groups. Level 5 activities are those where individuals engage in helping others in their social network, such as being a caregiver for a family member or a friend. Lastly, level 6 activities are types of social participation in which the individual contributes to society at large, including participating in political events or volunteering.

Using Levasseur’s model as the starting point, we categorized social activities into two groups: socializing activities and activities to help others. Socializing activities correspond to level 4 activities in the Levasseur’s framework and include (1) watching TV, listening to radio, music, reading newspaper or books *with family or friends*, (2) interacting with friends online (through email, online game, or electronic message), (3) playing cards, chess, or mahjong with others, (4) chatting, having tea, or hanging out with friends, (5) doing gardening with others, (6) taking a walk with others, (7) doing outdoor activities with others, (8) joining group exercise, and (9) participating in any religious groups, business groups, or seniors clubs. We coded someone as participating in socializing activities (“yes”) if they responded to ever having participated in any of the ten activities mentioned above and “no” otherwise.

The second type of social activity is one in which participants help others or contribute to society (labeled as “helping” activities). These activities correspond to the fifth and sixth levels of the Levasseur framework and include volunteering or joining political parties or campaigns. We coded someone as participating in helping activities (“yes”) if they had been a volunteer or joined any political parties or campaigns and “no” otherwise.

There are eight possible combinations of one’s socializing activities, helping activities, and working status in any survey wave. We coded each participant’s state as follows: (1) State A: helping, working, and socializing, (2) State B: helping and working, (3) State C: helping and socializing, (4) State D: working and socializing, (5) State E: socializing only, (6) State F: working only, (7) State G: helping only, and (8) State H: not involved in any of the social participation types. Each participant was labeled by a state in each wave, and we linked a participants’ states over the five waves to form a sequence, which was the basis of the sequence analysis described in the statistical analysis section below.

**Appendix B: Sensitivity Analysis**

The clustering results obtained from using the dataset with observed information on social participation and working states (n = 1,109) and the imputed dataset (n = 1,900) for the middle-aged subgroup are presented in Appendix Figures B.1. The clustering results were similar when comparing the two datasets. Appendix Table B.1 summarizes the association between social participation clustering and cognitive functioning in 2015 using OLS regression and censoring weights based on both the observed and the imputed datasets for the middle-aged subgroup. Cluster 3 (Working) and Cluster 4 (Active in multiple states) were significantly associated with higher cognitive function scores in 2015 for the two datasets. On the other hand, Cluster 1 (Socializing & helping) and Cluster 5 (Socializing) were significantly associated with higher cognitive function scores in 2015 for the observed dataset but not the imputed dataset.

Appendix Figures B.2 present the clustering results obtained from using the dataset with observed information on social participation and working states (n = 746) and the imputed dataset (n = 2,500) for the older subgroup. The clustering results appeared to be similar when comparing the two datasets, except for Cluster 4 (Working & low social participation). The observed dataset had more participants in the working only state (State F) over time. Appendix B.2 summarizes the association between social participation clustering and cognitive functioning in 2015 using OLS regression and censoring weights based on both the observed and the imputed datasets for the older subgroup. Cluster 5 (Helping & socializing) was consistently associated with higher cognitive function scores in 2015 for the two datasets but not for Cluster 4 (Working & low social participation).

**Appendix Figure B.1. Clusters of social participation histories, younger subgroup (age 50-65 in 1996).^*^**

| **1.1. Non-missing dataset (n=1,109)** | **1.2. Imputed dataset (n=1,900)** |
| --- | --- |
|  |  |

**^*^** Note: Social participation state A: helping, working, and socializing; State B: helping and working; State C: helping and socializing; State D: working and socializing; State E: socializing only; State F: working only; State G: helping only; State H: did not involve in any of the social participation types.

**Appendix Table B.1. Association between social participation clustering and cognitive functioning in 2015 using OLS regression and censoring weights^*^, younger subgroup (age 50-65 in 1996).**

| **Cluster** | **Non-missing dataset (n=1,109)** | | **Imputed dataset (n=1,900)** | |
| --- | --- | --- | --- | --- |
|  | **Coef.** | **95% C.I.** | **Coef.** | **95% C.I.** |
| Cluster 2: Low social participation (Ref)  Cluster 1: Socializing & helping  Cluster 3: Working  Cluster 4: Active in multiple states  Cluster 5: Socializing  Cluster 6: Helping | –  **0.86**  **1.02**  **1.27**  **0.59**  0.35 | –  **(0.32 – 1.39)**  **(0.40 – 1.65)**  **(0.74 – 1.80)**  **(0.06 – 1.12)**  (-0.19 – 0.89) | –  0.49  **0.52**  **0.58**  0.17  0.06 | –  (-0.002 – 0.98)  **(0.03 – 1.01)**  **(0.09 – 1.06)**  (-0.33 – 0.67)  (-0.46 – 0.59) |

^*^Adjusted for marital status, educational attainment, urbanicity of residence, medical diagnosis (cancer, hypertension, diabetes), baseline CESD score, health behaviors (smoking, drinking, betel nut use, regular exercise), number of grandchildren.

**Appendix Figure B.2. Clusters of social participation histories, older subgroup (age 65+ in 1996).^†^**

| **2.1. Non-missing dataset (n=746)** | **2.2. Imputed dataset (n=2,500)** |
| --- | --- |
|  |  |

**^†^** Note: Social participation state A: helping, working, and socializing; State B: helping and working; State C: helping and socializing; State D: working and socializing; State E: socializing only; State F: working only; State G: helping only; State H: did not involve in any of the social participation types.

**Appendix Table B.2. Association between social participation clustering and cognitive functioning in 2015 using OLS regression and censoring weights^†^, older subgroup (age 65+ in 1996).**

| **Cluster** | **Non-missing dataset (n=746)** | | **Imputed dataset (n=2,500)** | |
| --- | --- | --- | --- | --- |
|  | **Coef.** | **95% C.I.** | **Coef.** | **95% C.I.** |
| Cluster 3: Low social participation (Ref)  Cluster 1: Socializing  Cluster 2: Helping  Cluster 4: Working & low social participation  Cluster 5: Helping & socializing  Cluster 6: Active in multiple states | –  **1.10**  0.98  0.88  **1.62**  **2.07** | –  **(0.19 – 2.00)**  (-0.37 – 2.33)  (-0.19 – 1.95)  **(0.27 – 2.97)**  **(0.91 – 3.23)** | –  0.32  0.39  **2.33**  **1.15**  -0.32 | –  (-0.48 – 1.12)  (-0.78 – 1.55)  **(1.31 – 3.36)**  **(0.05 –2.26)**  (-1.24–0.60) |

**^†^** Adjusted for marital status, educational attainment, urbanicity of residence, medical diagnosis (cancer, hypertension, diabetes), baseline CESD score, health behaviors (smoking, drinking, betel nut use, regular exercise), number of grandchildren.

**Appendix C. Inverse probability of censoring weightings (IPCWs) method for the OLS model for the association between cluster membership and cognitive function in 2015.**

The weight ($IPCWi)$ for individual $i$ was calculated as follows:

$IPCW_{i}=\frac{\Pr\left[ D_{i}=0 | {A=A}_{i} \right]}{\Pr\left[ D_{i}=0 | {{A=A}_{i}, C=C}_{i} \right]}$ Eq. (A.1)

The denominator was the probability of observing an individual’s cognitive function score ($D_{i}=0)$ at the follow-up wave in 2015, conditional on his/her social participation cluster membership (${A=A}_{i}$) and time-invariant covariates ($C=C_{i})$, including sex, level of educational attainment, age, baseline CESD score, baseline urbanicity of residence, baseline number of grandchildren, baseline medical history (cancer, hypertension, and diabetes), and baseline health behaviors (smoking, drinking, betel nut use, regular exercise). The nominator was the probability that an individual’s cognitive function score was uncensored at the follow-up wave in 2015, conditional on his/her social participation cluster membership.
